# Prevalence of anxiety and depression following small subcortical ischemic stroke and their association with MRI-defined brain damage: The DHU-LAC cohort study

**DOI:** 10.1016/j.cccb.2025.100521

**Published:** 2025-11-25

**Authors:** Ana Dimitrovic, Aude Jabouley, Carla Machado, Marie-Helene de Sanctis, Annael Guedj, Lina Grosset, Antoine Guillonnet, Ruben Tamazyan, Joseph Benzakoun, Antoine Dusonchet, Catherine Oppenheim, Mathieu Zuber, David Calvet, Eric Jouvent

**Affiliations:** a APHP, Lariboisière Hospital, Department of Neurology and FHU NeuroVasc2030, F-75475 Paris, France; b APHP, Lariboisière Hospital, Department of Neuroradiology, F-75475 Paris, France; c Saint-Joseph Hospital, Department of neurology, Paris, France; d GHU-Paris Psychiatrie et Neurosciences, Sainte Anne Hospital, Department of neuroradiology, F-75014 Paris, France; e Institute of Psychiatry and Neuroscience of Paris (IPNP), INSERM U1266, IMA-Brain, 75014 Paris, France; f GHU-Paris Psychiatrie et Neurosciences, Sainte Anne Hospital, Department of neurology, F-75014 Paris, France; g Institute of Psychiatry and Neuroscience of Paris (IPNP), INSERM U1266; h Université Paris Cité, France

**Keywords:** Cognitive impairment, Minor stroke, MRI, Cerebral small vessel disease, Lacunes, White matter hyperintensities, Anxiety, Post-stroke depression

## Abstract

**Background:**

Data on imaging predictors of anxiety and depression remain limited in patients with minor stroke. We aimed to evaluate their prevalence six months after minor stroke and to determine whether MRI markers can help identify patients at increased risk.

**Methods:**

DHU-LAC is an ongoing multicentre prospective French study including patients with minor ischaemic stroke likely due to cerebral small vessel disease. Anxiety and depression were assessed at baseline and 6 months using the Hospital Anxiety and Depression Scale (HADS-A and HADS-D; scores≥8 indicating mild, ≥11 moderate symptoms). Disability was measured with the modified Rankin Scale (mRS), global cognition with the Montreal Cognitive Assessment (MOCA), and quality of life with the EQ-5D. Brain damage on MRI was assessed via the CSVD score. Predictors of HADS-A and HADS-D were explored using linear mixed-effects models.

**Results:**

Among 163 participants (mean age 63 ± 11 years; 28 % women), residual disability was minimal at 6 months (median mRS [interquartile range]: 1 [0.5–1.5]). At that time, 40 % of participants showed mild to moderate anxiety scores, and 19 % mild to moderate depression scores. In multivariable models, lower anxiety and depression scores were associated with better EQ-5D (β=–0.045, *p* < 0.001; β =–0.13, *p* = 0.021 respectively) and MOCA (β=–0.058, *p* < 0.001; β=–0.11, *p* = 0.028) scores. No consistent clinical or imaging predictors of elevated anxiety or depression scores were identified.

**Conclusions:**

Anxiety and depression are common after minor stroke despite minimal physical disability. The absence of clear imaging or clinical predictors highlights the importance of systematic psychological screening, even in patients with minor stroke.

## Highlights

• We found a high prevalence of anxiety and depression in a clinically homogeneous cohort of patients with minor stroke.
• Lower levels of anxiety and depression—even among patients who had achieved near-complete recovery—were still associated with better cognitive outcomes and higher perceived quality of life.
• Despite the homogeneity of this cohort, no imaging markers were identified as significant predictors of elevated anxiety or depression six months after stroke.

## 1 Introduction

Stroke is a leading cause of death and disability worldwide [1]. The consequences of stroke are heterogenous and depend on multiple factors, possibly including the location and severity of brain tissue damage [2]. While some patients achieve full recovery, others remain severely disabled. Beyond physical impairments, stroke often results in cognitive deficits, communication difficulties, emotional and behavioural disturbances, pain, and fatigue, all of which can markedly reduce quality of life.

It has long been proposed that the prevalence of neurobehavioral disturbances, including anxiety and depression, increases with stroke severity [[3], [4], [5]]. Several studies have investigated neurobehavioral alterations following minor stroke; however, most share similar methodological limitations, resulting in either very small or highly heterogeneous samples. Many included patients with transient ischemic attacks (TIAs), some of whom were later reclassified as minor strokes based on imaging findings, occasionally with remote post-event assessments [[6], [7], [8]]. Most studies did not distinguish between ischemic and haemorrhagic strokes and included patients with cortical lesions, potentially leading to symptoms such as aphasia or neglect that can interfere with the assessment of neurobehavioral outcomes [[9], [10], [11]]. Neurobehavioral alterations have also been reported in the pivotal SPS3 trial, which incorporated imaging-based inclusion criteria; however, patients were primarily enrolled during the secondary prevention phase, up to six months after the initial stroke event [12].

In the present study, we aimed to assess anxiety and depression scores in a highly homogeneous cohort of patients who were all hospitalized in a stroke unit for a small subcortical ischemic stroke confirmed by MRI, enrolled within 15 days of the event, and evaluated after 6 months. This design provides a unique opportunity to test the hypothesis that brain imaging markers can help identify, within this specific population, patients at higher risk of developing anxiety and depression after stroke.

## 2 Material and methods

### 2.1 Study population

DHU-LAC is an ongoing prospective French multicentre cohort intended to recruit 500 patients with ischemic stroke presumably related to cerebral small vessel disease (cSVD). Patients are recruited in the stroke units of four academic hospitals in Paris area, France: Lariboisière, Bichat, Sainte-Anne and Saint-Joseph (Clinical trials number: NCT03552926). As Bichat began recruitment after the inclusion of the participants of the present study, data and co-authors are not associated with the present paper. Since June 2018, consecutive patients presenting within 15 days of stroke onset, with brain MRI demonstrating a recent small subcortical infarct (STRIVE2 criteria) [13], explaining the symptoms, and willing to participate, are included. Details regarding inclusion and exclusion criteria are provided elsewhere (https://clinicaltrials.gov) and in the supplementary material. Noteworthily, pre-stroke disability (modified Rankin scale (mRS) ≥ 4) and major neurocognitive disorder according to DSMIV criteria are part of exclusion criteria. Systematic work-up, with cardiac investigations, blood sampling, imaging of head and neck vessels, is performed during the acute phase according to the centres’ daily clinical practice. Additional tests, such as additional explorations to detect atrial fibrillation or a cancer work-up, are carried out when deemed necessary by the team in charge, but patients with such causes are not excluded. Secondary prevention medications are prescribed in accordance with current international guidelines. As recruitment criteria involves a small sized subcortical lesion, patients initially present with low to moderate motor symptoms, and without language alterations, making of DHU-LAC a valuable minor stroke cohort to evaluate remote neurobehavioral consequences of stroke. In DHU-LAC, patients are systematically evaluated 6 months after the stroke, clinically, with a comprehensive neuropsychological battery, and with a standardized brain MRI. They then undergo annual clinical follow-up for up to 5 years (data not yet available).

### 2.1 Clinical evaluation

Among other parameters, stroke severity is evaluated with the NIH stroke scale (NIHSS) and disability with the modified Rankin’s scale (mRS) at baseline and after 6 months and yearly afterwards. Age, sex and level of education are recorded at inclusion. History of psychiatric disorder is defined as a history of psychiatric follow-up, hospitalization for psychiatric reasons or taking corresponding medications.

### 2.2 Neuropsychological testing and clinical evaluation

As previously detailed [14], patients are evaluated by an experienced neuropsychologist at baseline and after 6 months. Only the following tests performed at both occasions are used in the present study: the Hospital Anxiety and Depression Scale (HADS) [15,16], rated separately for anxiety and depression subscales, with widely accepted cut-offs for both: normal 0–7, borderline abnormal 8–10 and abnormal 11–21 [16,17]); the MOCA (Montreal cognitive assessment test) as an screening tool of global cognitive performances [18]; and the EQ-5D questionnaire, by which respondents can report their perceived health related quality of life with a grade ranging from 0 (worst) to 100 (best) [19]. In addition, pre-stroke functional status is assessed using the short form of the Informant Questionnaire on Cognitive Decline in the Elderly (IQCODE-R) [20], together with evaluations of daily functioning through the Activities of Daily Living (ADL) [21] and Instrumental Activities of Daily Living (IADL) [22] scales.

### 2.3 Magnetic resonance imaging, MRI markers, and post-processing

Prior to recruitment, the index ischemic lesion is identified on a non-standardized brain MRI performed in the acute phase. Although imaging parameters and acquisition settings may vary across centres—particularly as scans are sometimes performed at night or in external hospitals before referral to the recruiting site—the protocol in all cases includes at least a diffusion-weighted sequence with ADC and B1000 mapping, an axial Fluid-Attenuated Inversion Recovery (FLAIR) sequence, and a magnetic susceptibility sequence (2D T2* on 1.5T scanners and SWI on 3T scanners). In contrast, the 6 month follow-up MRI scans are performed with predefined protocols, including high-resolution 3D gradient echo T1-weighted sequences, high-resolution 3D Fluid-Attenuated Inversion Recovery (FLAIR) sequences, diffusion sequence and SWI sequences [14].

Lacunes, white matter hyperintensities and microbleeds are defined according to the STRIVE2 criteria [23], and rated blind to clinical information on the 6 month MRI scans. Lacunes are rated manually by an expert with strong experience in lacune identification [24]. Fazekas scale is rated on the FLAIR sequence, and the number of microbleeds on the SWI sequence. For all patients, we determined the total CSVD score, a simple composite metric reflecting the severity of the most established MRI markers of cSVD. One point is assigned for the presence of lacunes, extensive white matter hyperintensities, microbleeds, and severe dilated perivascular spaces in the basal ganglia, as previously described, yielding a total score ranging from 0 to 4 to capture overall cSVD burden [25]. In the context of DHU-LAC, in which patients are recruited based on the morphology of the index ischemic stroke lesion independent of the identification during the work-up of associated causes, CSVD score also reflects the likelihood of the stroke being actually related to cSVD. We used the TOAST classification to illustrate this point [26]. For each patient, we determined the location of the index stroke event lesion (thalamus, basal ganglia, internal capsule, brainstem, other), in line with the hypothesis that some strategic areas may be more prone to be associated with anxiety and depression [27].

### 2.4 Statistical analysis

Statistical analyses were performed with R (version 4.4.2). The mean ± standard deviations were used to describe normal variables, median with interquartile range otherwise. Numbers and percentages are given in tables for categorical variables. Differences in categorical variables between groups were assessed using the Chi-square (verified with Fisher’s exact test for small cell counts). Continuous variables were compared using the Wilcoxon rank sum test for non-normally distributed data. Differences in the distribution of categorical subtypes (e.g., lesion location) between groups were evaluated using the Chi-square test of homogeneity. Details are provided in the tables. To evaluate the impact of the different variables on anxiety and depression scores over time, we built linear mixed models to predict anxiety and depression scores respectively while taking into account repeated measures. The model’s assumptions, including the normality of residuals, were systematically checked. We included systematically as covariates those that were significant in first line group comparisons based on the literature as well as the CSVD score. We used the LMER package from R. Significance level was set at 0.05 two-sided.

### Standard protocol approvals, registrations, and patient consents

Ethics committee agreement was obtained for DHU-LAC cohorts (authorization IRB00003835 - ref 2015/47). Written informed consent was obtained from all participants.

## 3 Results

At time of October 17^th^, 2023, 249 patients had been included, among whom 23 had not yet reached the end of the 6-month follow-up period and were not considered in analyses. Fifty-four patients (24 %) reached the end of the 6-month follow-up but had no complete follow-up evaluation: 6 died; 2 become too disabled to be evaluated; 15 did not attend the 6-month evaluation and had further clinical evaluations since then; 15 withdrew their consent; 16 were lost to follow-up. Nine patients did not have their HADS evaluation: 2 were too exhausted, 1 was too depressed and the reason for unavailability of data was unknown for 7. Hence, the present analyses correspond to 163 patients at the six months follow-up with complete neuropsychological evaluation.

The mean time to baseline neuropsychological evaluation was 4 days after the stroke, and that to the comprehensive battery was 7.1 ± 2.2 months. An informant was available, allowing the IQCODE-R to be obtained for 93 of the 166 participants. Among these, 91 % had an IQCODE-R score below 3.4. ADL and IADL data were available for all but two patients. Full scores (8/8 for IADL and 6/6 for ADL) were observed in 84 % and 83 % of participants, respectively, with an interquartile range of 0 for both measures. Characteristics of the patients are detailed in Table 1. Twenty-eighth percent of patients were women. Median age was 63 years. Only 7 % of patients had a pre-stroke history of psychiatric disorder.

**Table 1:** **Baseline and 6-month characteristics.** Characteristics of the study sample at baseline and six months. Abbreviations: NIHSS, National Institutes of Health Stroke Scale; mRS, Modified Rankin Scale; HADS, Hospital Anxiety and Depression Scale; with anxiety and depression subscales. CSVD: Cerebral small vessel disease score.

***N* = 163^1^**		
	**Baseline**	**6-month follow-up**
Female	46 (28 %)	
Age (years)	63 (56 – 70)	
History of psychiatric disorder	11 (7 %)	
HADS-A ≥ 8 ≤ 10 (mild anxiety) ≥ 11 (moderate anxiety)	39 (24 %) 24 (15 %)	31 (19 %) 34 (21 %)
HADS-D ≥ 8 ≤ 10 (mild depression) ≥ 11 (moderate depression)	18 (11 %) 7 (4 %)	22 (13 %) 10 (6 %)
NIHSS		0 (0 – 0)
mRS		1 (0.5 – 1.5)
		
**TOAST Classification**		
- Large artery atherosclerosis		11 (7 %)
- Cardioembolic		11 (7 %)
- cSVD		87 (53 %)
- Other determined cause		2 (1 %)
- Undetermined cause		52 (32 %)
		
**Imaging**		
Index stroke lesion location		
- Thalamus		46 (28 %)
- Basal ganglia		26 (16 %)
- Internal capsule		32 (20 %)
- Brainstem		38 (23 %)
- Other		21 (13 %)
Left side lesion		83 (51 %)
Fazekas scale		
- 0		25 (15 %)
- 1		57 (35 %)
- 2		44 (27 %)
- 3		37 (23 %)
Presence of lacunes		70 (43 %)
Presence of microbleeds		47 (29 %)
Presence of severe dilation of perivascular spaces		57 (35 %)
CSVD score		
- 0		45 (28 %)
- 1		23 (14 %)
- 2		44 (27 %)
- 3		26 (16 %)
- 4		25 (15 %)

N ( %); Median (IQR).

Patients had a low level of disability at baseline and follow-up (median NIHSS and mRS at 0 and 1 respectively at 6 months). At 6 months, anxiety and depression (either mild and moderate levels according to the HADS) were noted in 40 % and 19 % of patients respectively.

Participants with mild or moderate anxiety scores at 6 months were more frequently female and had lower levels of education (Table 2). They also exhibited significantly greater disability, reduced global cognitive performance, and poorer quality of life. No significant association was observed with social isolation, although a trend was noted for a history of psychiatric disorders. Patients with high anxiety scores at 6 months generally also had high anxiety scores at baseline.

**Table 2:** **Patient characteristics according to HADS anxiety subscale categories at 6 months.** Comparisons of patient characteristics according to anxiety levels on the HADS (none, mild, moderate) assessed at the 6-month visit. Abbreviations: NIHSS, National Institutes of Health Stroke Scale; mRS, Modified Rankin Scale; MoCA, Montreal Cognitive Assessment.

**Categories of HADS anxiety subscale at 6 months**					
	< 8 (no anxiety) (*n* = 98)	8–10 (mild anxiety) (*n* = 31)	≥11 (moderate anxiety) (*n* = 34)	p-value (no vs mild)	p-value (no vs moderate)
**Epidemiological factors at baseline**					
Female ^§^	21 (21 %)	11 (35 %)	16 (47 %)	0.13	**<0.001**
Level of education ≥12 years ^§^	52 (53 %)	13 (43 %)	11 (33 %)	0.24	**0.04**
Single, widowed or divorced ^§^	31 (32 %)	10 (32 %)	11 (32 %)	0.94	0.27
History of psychiatric disorder ^§^	3 (3 %)	4 (13 %)	4 (13 %)	0.13	0.13
**Clinical and neurobehavioral variables at baseline**					
NIHSS ^†^	1 (0–2)	1 (0–2)	1 (0–2)	0.13	0.13
mRS ^†^	1 (0.5–1.5)	1 (0.5–1.5)	1.5 (1–2)	0.23	0.12
HADS anxiety subscale ^#^				**<0.001**	**<0.001**
- ≥ 8 ≤ 10 (mild)	13 (13 %)	15 (47 %)	28 (82 %)		
- ≥ 11 (moderate)	4 (4 %)	3 (10 %)	16 (49 %)		
HADS depression subscale ^#^				**<0.001**	**0.004**
- ≥ 8 ≤ 10 (mild)	6 (6 %)	6 (20 %)	10 (30 %)		
- ≥ 11 (moderate)	2 (2 %)	2 (7 %)	3 (9 %)		
**Clinical and neurobehavioral variables at the 6-month visit**					
NIHSS ^†^	0 (0–0)	0 (0.5–1.5)	0 (0–1)	0.09	0.13
mRS ^†^	0 (0–1)	1 (0–2)	1 (0.5–1.5)	**0.03**	**0.009**
MoCA ^†^	26 (24–28)	22 (19–25)	24 (21–27)	**0.004**	**0.008**
EQ-5D VAS ^†^	80 (70–90)	60 (47.5–72.5)	60 (45–75)	**<0.001**	**<0.001**
HADS-D categories ^#^				**0.001**	**<0.001**
- ≥8 ≤ 10 (mild depression)	24 (37 %)	6 (19 %)	16 (47 %)		
- ≥11 (moderate depression)	10 (15 %)	2 (6 %)	8 (24 %)		
					
TOAST Classification ^#^					
- Large artery atherosclerosis	9 (9 %)	1 (3 %)	1 (3 %)	0.71	0.58
- Cardioembolic	8 (8 %)	1 (3 %)	2 (7 %)		
- cSVD	47 (48 %)	20 (65 %)	20 (57 %)		
- Other determined cause	2 (2 %)	0	0		
- Undetermined cause	32 (33 %)	9 (29 %)	11 (33 %)		
					
**Radiological data at 6 months**					
Index stroke lesion location ^#^					
- Thalamus	28 (29 %)	13 (41 %)	5 (15 %)	0.17	0.14
- Basal ganglia	14 (14 %)	5 (19 %)	7 (19 %)		
- Internal capsule	21 (21 %)	5 (14 %)	6 (16 %)		
- Brainstem	22 (22 %)	5 (17 %)	11 (32 %)		
- Other	13 (14 %)	2 (7 %)	6 (18 %)		
Left side lesion ^§^	47 (48 %)	17 (54 %)	19 (56 %)	0.71	0.59
Fazekas scale ^#^					
- 0	17 (17 %)	4 (13 %)	4 (11 %)	0.33	0.17
- 1	38 (39 %)	11 (37 %)	8 (25 %)		
- 2	23 (23 %)	6 (19 %)	15 (44 %)		
- 3	21 (21 %)	9 (31 %)	7 (20 %)		
Presence of lacunes ^§^	43 (44 %)	13 (45 %)	14 (37 %)	0.93	0.47
Presence of microbleeds ^§^	27 (26 %)	11 (39 %)	9 (23 %)	0.35	0.71
Presence of severe dilation of perivascular spaces ^§^	34 (35 %)	10 (36 %)	13 (37 %)	0.78	0.69
CSVD score ^#^					
- 0	29 (30 %)	10 (34 %)	6 (19 %)	0.56	0.36
- 1	18 (18 %)	1 (2 %)	4 (11 %)		
- 2	23 (23 %)	6 (22 %)	15 (43 %)		
- 3	13 (13 %)	7 (23 %)	6 (17 %)		
- 4	16 (16 %)	6 (19 %)	3 (10 %)		

N ( %); Median (IQR); ^†^ non-normally distributed continuous variables compared using the Wilcoxon rank-sum test; ^§^ categorical variables compared using the Chi-square test; ^#^ distribution of proportions across categories compared using the Chi-square test of homogeneity.

Participants with mild or moderate depression scores at 6 months were also more likely to present with disability and lower quality of life and to have a history of psychiatric disorders (see Table 3 for details). Participants with high depression scores at 6 months had already exhibited high depression scores at baseline.

**Table 3:** **Patient characteristics according to HADS depression subscale categories at 6 months.** Comparisons of patient characteristics according to depression levels on the HADS (none, mild, moderate) assessed at the 6-month visit. Abbreviations: NIHSS, National Institutes of Health Stroke Scale; mRS, Modified Rankin Scale; MoCA, Montreal Cognitive Assessment.

**Categories of HADS depression subscale at the 6-month visit**					
	< 8 (no depression) (*n* = 131)	8–10 (mild depression) (*n* = 22)	≥11 (moderate depression) (*n* = 10)	p-value (no vs mild)	p-value (no vs moderate)
**Epidemiological factors**					
Female ^§^	35 (27 %)	6 (29 %)	5 (50 %)	0.8	0.2
Level of education ≥12 years ^§^	67 (51 %)	8 (35 %)	2 (22 %)	0.7	0.07
Single, widowed or divorced ^§^	41 (31 %)	8 (38 %)	3 (33 %)	0.15	0.88
History of psychiatric disorder ^§^	4 (3 %)	4 (20 %)	2 (20 %)	0.1	0.3
**Clinical and neurobehavioral variables at baseline**					
NIHSS ^†^	1 (0–2)	2 (1.5–2.5)	3(1.5–4.5)	0.09	0.1
mRS ^†^	1 (0.5–1.5)	1 (0.5–1.5)	2 (1–3)	0.3	0.06
HADS anxiety subscale ^#^				**0.1**	**0.04**
- ≥ 8 ≤ 10 (mild)	11 (9 %)	2 (10 %)	5 (50 %)		
- ≥ 11 (moderate)	2 (2 %)	7 (30 %)	4 (40 %)		
HADS depression subscale ^#^				**0.001**	**0.04**
- ≥ 8 ≤ 10 (mild)	41 (31 %)	8 (35 %)	8 (80 %)		
- ≥ 11 (moderate)	14 (11 %)	3 (15 %)	2 (20 %)		
**Clinical and neurobehavioral variables at the 6-month visit**					
NIHSS ^†^	0 (0–0)	0 (0–0)	1 (0–2)	0.5	0.1
mRS ^†^	1 (0.5- 1.5)	1 (0.5–1.5)	2 (1.5–2.5)	0.3	**0.01**
MoCA ^†^	26 (24–28)	25 (24–26)	22 (21–23)	0.7	0.1
EQ-5D VAS ^†^	75 (62.5- 87.5)	65 (55–75)	37.5 (28.5- 46.5)	**0.01**	**<0.001**
HADS-D categories ^#^				**0.002**	**<0.001**
- ≥8 ≤ 10 (mild depression)	40 (31 %)	6 (29 %)	10 (100 %)		
- ≥11 (moderate depression)	17 (13 %)	7 (33 %)	8 (80 %)		
					
TOAST Classification ^#^					
- Large artery atherosclerosis	10 (7.5 %)	1 (5 %)	0	0.47	0.15
- Cardioembolic	11 (9 %)	0	0		
- cSVD	68 (52 %)	14 (62 %)	5 (50 %)		
- Other determined cause	2 (1.5 %)	0	0		
- Undetermined cause	40 (30.5 %)	7 (33 %)	5 (50 %)		
					
**Radiological data at 6 months**					
Index stroke lesion location ^#^					
- Thalamus	37 (28 %)	8 (35 %)	1 (14 %)	0.08	0.14
- Basal ganglia	20 (15 %)	6 (24.5 %)	0		
- Internal capsule	26 (19.5 %)	5 (24.5 %)	1 (14 %)		
- Brainstem	31 (23.5 %)	3 (16 %)	4 (43 %)		
- Other	18 (14 %)	0	3 (29 %)		
Left-side lesion	65 (49 %)	12 (53 %)	6 (57 %)	0.75	0.69
Fazekas scale ^#^					
- 0	19 (14 %)	4 (19 %)	2 (25 %)	0.83	0.06
- 1	48 (36.5 %)	7 (33 %)	2 (25 %)		
- 2	33 (25 %)	6(28 %)	5 (50 %)		
- 3	32 (24.5 %)	5 (20 %)	0		
Presence of lacunes ^§^	53 (40 %)	9 (43 %)	8(75 %)	0.81	0.07
Presence of microbleeds ^§^	40 (30 %)	2 (10 %)	5 (50 %)	0.02	0.43
Presence of severe dilation of perivascular spaces ^§^	49 (37.5 %)	3 (14 %)	5 (50 %)	0.01	0.54
CSVD score ^#^					
- 0	38 (29 %)	7 (33 %)	0	0.25	0.06
- 1	19 (14 %)	3 (14 %)	1 (12 %)		
- 2	32 (24 %)	8 (34 %)	4 (38 %)		
- 3	19 (15 %)	4 (19 %)	3 (25 %)		
- 4	23 (18 %)	0	2(25 %)		

N ( %); Median (IQR); ^†^ non-normally distributed continuous variables compared using the Wilcoxon rank-sum test; ^§^ categorical variables compared using the Chi-square test; ^#^ distribution of proportions across categories compared using the Chi-square test of homogeneity.

We found no consistent associations between the presence of mild or moderate anxiety and/or depression scores at 6 months and imaging findings, whether related to the index stroke lesion or to remote cSVD–related damage (see Table 2, Table 3). Two p-values reached nominal significance (*p* < 0.05) for associations between mild depression and the presence of microbleeds and dilated perivascular spaces.

To better understand the determinants of anxiety and depression scores over time, we used linear longitudinal mixed-effect models including systematically as covariates age, sex, level of education, MOCA, mRS, history of psychiatric disorder, EQ-5D as suggested by raw group comparisons, and CSVD score. We found that higher anxiety scores over time are significantly associated with higher depression scores (β = 0.26, *p* < 0.001), with a strong bidirectional relationship (β = 0.24, *p* < 0.001). Anxiety scores were significantly higher in women (β = 1.22, *p* = 0.017) and tended to be higher in patients with a history of psychiatric disorders (β = 1.66, *p* = 0.070). In contrast, anxiety scores were lower in participants with higher perceived quality of life on the EQ-5D and better scores at the MOCA (β = –0.045, *p* < 0.001 and β = –0.13, *p* = 0.021 respectively). No significant relationships were observed for age, level of education, CSVD score or mRS.

In line, participants with higher depression scores over time had significantly more frequently a history of psychiatric disorder (β = 1.84, *p* = 0.028). Participants with lower depression scores had significantly higher MOCA scores and higher EQ-5D scores (β = –0.11, *p* = 0.028 and β = –0.058, *p* < 0.001 respectively), and women tended to show lower depression scores (β = –0.85, *p* = 0.066). In contrast, age, level of education, CSVD scores and mRS were not significantly associated with scores of depression.

## 4 Discussion

In this prospective multicentre French cohort of patients with minor stroke—characterized by very low initial severity and minimal residual disability—all of whom were hospitalized in a stroke unit and enrolled within 15 days of the event following MRI confirmation of an acute subcortical ischemic lesion, we observed a high prevalence of mild to moderate anxiety and depression scores at both baseline and at the 6-month follow-up assessment.

As expected, higher anxiety and depression scores were strongly correlated. Higher levels of both anxiety and depression were significantly and independently associated with lower perceived quality of life. Although the directionality of this relationship cannot be determined in this observational context, these findings highlight the central role of quality of life in post-stroke emotional well-being and patient management. Higher anxiety and depression scores were also consistently associated with worse overall cognitive performance, as measured—albeit imperfectly—by the MOCA. This contrasts with some previous reports, which may reflect differences in patient recruitment strategies or sample characteristics [28]. Conversely, we found no significant association between anxiety and depression scores and several potential determinants, including disability or level of education. Associations with female sex and a history of psychiatric disorders were inconsistent across anxiety and depression measures, which is generally in line with prior literature [29]. Importantly, the high prevalence of elevated anxiety and depression scores observed in our cohort is consistent with proportions reported in markedly different contexts, including TIA and/or minor stroke populations [8,11,30] as well as in cSVD [12]. These proportions appear to be of the same order of magnitude as those reported in unselected stroke populations [31]. Comparisons with patients from other French cohorts also revealed similar profiles [32,33].

Taking advantage of the homogeneity of the DHU-LAC cohort, we tested whether specific imaging markers could predict the risk of elevated anxiety and depression scores during follow-up, but no significant associations were found. Neither raw correlations nor linear mixed-model analyses assessing longitudinal changes in anxiety and depression scores provided evidence of relationships between lesion location, the presence of remote lesions in the context of cSVD, or any other imaging parameter. We identified two p-values below 0.05 indicating associations between depression and the presence of microbleeds and dilated perivascular spaces; however, these findings were inconsistent and most likely reflect chance effects related to multiple testing, which we chose not to correct for. Our findings support the hypothesis that the absence of associations between imaging and neurobehavioral symptoms likely reflects a true lack of causal relationship rather than limitations related to sample heterogeneity in previous studies. This further reinforces the notion that all stroke patients, regardless of lesion characteristics, may be at risk.

Our study has some major strengths. First, all patients were included after a brain MRI demonstrated an ischaemic lesion corresponding to the stroke, while minor stroke cohort may be otherwise subject to diagnostic uncertainty when significant proportions of patients have a normal CT scan and the diagnosis is purely clinical. The DHU-LAC cohort is characterised by recruitment based on lesion morphology and independent of the causal disease, which is deliberate as there is currently no recognised diagnostic method to guarantee that a small subcortical infarct is actually due to cSVD. With this in mind, the DHU-LAC cohort includes patients who are homogeneous in terms of eligible index lesion but heterogeneous in terms of underlying cause and degree of remote brain damage. This allowed us to observe that the event itself, by confronting relatively young patients with the risk of death and disability, may well be the main factor contributing to anxiety and depression. Another key aspect related to the inclusion of only small subcortical ischemic stroke is the absence of language disturbance in our sample, which could be leading to both anxiety and depression per se, and also a strong confounding factor when evaluating cognitive and behavioural post-stroke alterations.

Our study also has several limitations. First, to assess depression and anxiety, we relied on scores rather than clinical diagnoses. However, our study shares this limitation with most other studies in this field. We must also acknowledge a possible significant underestimation of these scores. Indeed, a non-negligible number of patients were not assessed. These patients are likely to have higher anxiety and depression scores than those for whom complete evaluations were obtained. Pre-stroke assessment measures were also limited. The IQCODE-R was included in the participants’ evaluation; however, due to the strict inclusion criteria resulting in the enrolment of patients in generally good health, investigators often faced difficulties contacting informants. Consequently, the IQCODE-R score was available for only 93 of 166 patients. In contrast, the Instrumental Activities of Daily Living (IADL) and Activities of Daily Living (ADL) indices showed a high level of pre-stroke autonomy. Unfortunately, fatigue—although a major issue for patients—is not assessed in the DHU-LAC cohort. We did not systematically adjust for multiple comparisons, as we chose to construct a single multivariable predictive model for anxiety and depression. Given that our findings regarding anxiety and depression scores are consistent with the existing literature and that no consistent associations were observed with imaging variables, applying such an adjustment would not have altered the results. The selection of predictors for the anxiety and depression models was based on prior evidence and observed associations; nevertheless, this approach may have introduced bias, and the results should therefore be interpreted with caution. We mostly used the CSVD score to address the link with depression and anxiety, whereas much more powerful approaches could have been used. When more data becomes available, we intend to use more sophisticated approaches, including voxel-by-voxel statistical analyses. Finally, our sample comprises less than one-third-of women, a finding that we have previously reported and that we are currently investigating. To the best of our knowledge, this may suggest that female patients are less likely to be admitted in stroke units [14].

In conclusion, we report that high anxiety and depression scores are very common, both immediately after and 6 months after a minor stroke, even with minimal residual disability, as evaluated with widely used tools. As we identified no obvious predisposing factors, such as psychiatric history, previous brain damage, or epidemiological status, our results support large-scale screening for anxiety and depression. However, to obtain formal evidence of the usefulness of this recommendation, intervention trials would probably need to be conducted.

## Data availability

Anonymized data will be shared by request from any qualified investigator for the sole purpose of replicating procedures and results presented in the article and as long as data transfer is in agreement with EU legislation on the general data protection regulation.

## Funding

The study was funded by Assistance-Publique Hôpitaux de Paris, GHU Paris Neurosciences, and Saint-Joseph Hospital, together with FHU Neurovasc 2030.

## Declaration statement

I, Eric Jouvent, corresponding author of the manuscript entitled “**Prevalence of anxiety and depression after small subcortical ischemic stroke and the impact of brain damage: the DHU-LAC cohort”, hereby certify that:**

This work has not been published previously

It is not under consideration for publication elsewhere.

Its submission has been approved by all authors and tacitly or explicitly by the responsible authorities where the work was carried out. if accepted, the article will not be published elsewhere in the same form, in English or in any other language, including electronically, without the written consent of the copyright-holder.

## CRediT authorship contribution statement

**Ana Dimitrovic:** Writing – review & editing, Writing – original draft, Methodology, Investigation, Formal analysis, Data curation. **Aude Jabouley:** Writing – review & editing, Methodology. **Carla Machado:** Writing – review & editing, Methodology. **Marie-Helene de Sanctis:** Writing – review & editing, Methodology. **Annael Guedj:** Writing – review & editing, Methodology. **Lina Grosset:** Writing – review & editing, Methodology. **Antoine Guillonnet:** Writing – review & editing, Methodology. **Ruben Tamazyan:** Writing – review & editing, Methodology. **Joseph Benzakoun:** Writing – review & editing, Methodology, Investigation. **Antoine Dusonchet:** Writing – review & editing, Methodology, Investigation. **Catherine Oppenheim:** Writing – review & editing, Methodology, Funding acquisition, Conceptualization. **Mathieu Zuber:** Writing – review & editing, Methodology, Funding acquisition, Conceptualization. **David Calvet:** Writing – review & editing, Writing – original draft, Methodology, Funding acquisition, Conceptualization. **Eric Jouvent:** Writing – review & editing, Writing – original draft, Methodology.

## Declaration of competing interest

The authors declare no competing interests.

## Appendix Supplementary materials

Image, application 1
