# Supplementary material for: Prevalence of anxiety and depression following small subcortical ischemic stroke and their association with MRI-defined brain damage: The DHU-LAC cohort study
Source: Cereb Circ Cogn Behav. 2025 Nov 25;9:100521. doi: 10.1016/j.cccb.2025.100521 (PMC12719186; doi:10.1016/j.cccb.2025.100521)
Supplement: Supplementary file 1 [file mmc1.docx]

| **Inclusion** | **Exclusion** |
| --- | --- |
| **Age 18 years and above** | **Prior diagnosis of dementia according to DSM IV criteria** |
| **History of recent (less than 15 days) neurological deficit related to a small subcortical infarct (evidenced by medical history or physical examination)** | **Prior severe disability with Rankin scale ≥ 4** |
| **Diagnosis of recent small subcortical infarct on MRI (hyperintensity on diffusion-weighted imaging showing an infarction in the territory of one perforating artery) presumably responsible for the corresponding neurological deficit** | **Serious concomitant systemic disorder that can compromise the follow-up study** |
| **Lesion diameter mess than 20 mm** | **Leukoencephalopathy of non-vascular origin** |
| **Ability to comply with scheduled follow-up and annual neurological evaluation** | **Severe psychiatric disorder** |
| **Affiliation to the national French health insurance** | **Inability to obtain an informed signed consent from the patient or his/her family** |
| **Signature of informed consent** | **Medical contraindication or refusal to undergo cerebral magnetic resonance scanning (MRI)** |
